# Seasonal and Environmental Influences on Free Sugar and Amino Acid Profiles of *Lycium barbarum* Berries Cultivated in Southern Tuscany

**DOI:** 10.1002/fsn3.71568

**Published:** 2026-02-19

**Authors:** Letizia Poggioni, Giampiero Cai, Claudio Cantini, Marco Romi, Chiara Piccini

**Affiliations:** ^1^ Department of Life Sciences University of Siena Siena Italy; ^2^ National Research Council of Italy Institute for Bioeconomy (CNR‐IBE) Follonica Italy

**Keywords:** amino acids, environmental influences, *L. barbarum*, seasonal trends, soluble sugars

## Abstract

*Lycium barbarum* L. (Goji) berries are highly valued for their nutritional and nutraceutical properties, largely due to their sugar and amino acid content. This study analyzed organically cultivated berries from southern Tuscany (Italy), sampled across four ripening stages (July–November). Free sugars were measured over 3 years (2018–2020), and amino acids over two (2019–2020). Fully ripe berries (S4) exhibited high levels of glucose (80.2 ± 19.3 mg/g FW), fructose (75.5 ± 18.1 mg/g FW), and pectins (46.3 ± 16.1 mg/g FW), while sucrose remained low. Seasonal and interannual trends revealed progressive increases in glucose, fructose, pectins, and ethanol (up to 15.9 mg/g FW), positively correlated with precipitation and negatively with temperature. Total free amino acids averaged 3.21 mg/g FW, with non‐essential amino acids representing 55.4%. Proline was most abundant (1.05 mg/g FW), and its accumulation increased with rainfall and decreased with higher temperatures. Other compounds influenced by climatic stress included β‐aminobutyric acid (BABA) and ornithine. These findings highlight the strong impact of environmental variables on berry metabolism, particularly in sugar and amino acid biosynthesis. The optimal harvest window lies between version and full ripeness, when metabolic profiles peak. This work underscores the importance of climate‐driven strategies to optimize goji berry quality through informed cultivation practices.

## Graphical abstract

The graphical abstract illustrates how temperature, rainfall, and ripening stage shape the metabolic profile of *Lycium barbarum* berries grown in Southern Tuscany. It highlights the optimal harvest window (S2–S4) and summarizes key trends in sugars (glucose, fructose, pectins) and amino acids (total FAAs, proline, BABA, ornithine). Visual cues show that rainfall enhances, while higher temperatures reduce metabolite accumulation. Together, these elements convey the strong climate‐driven modulation of goji berry nutritional quality.

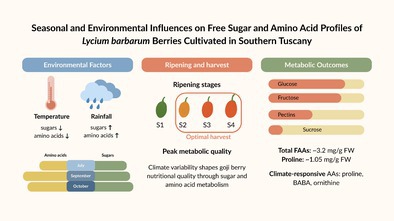

## 1 Introduction

In recent decades, global berry cultivation and production have increased markedly, driven by growing consumer demand and heightened awareness of their potential health benefits. This trend involves both widely known species, such as strawberries and blueberries, and increasingly includes more exotic fruits like goji berries. Wild and cultivated berries alike are well recognized as rich sources of bioactive compounds that have attracted significant scientific attention due to their potential health‐promoting and disease‐preventing properties. Among these compounds, polyphenols, vitamins, and dietary fiber stand out as key contributors to the distinctive nutritional profile of berries (Barkaoui et al. 2023; Beattie et al. 2005; Golovinskaia and Wang 2021). In particular, Goji berries contain carbohydrates (46%), fiber (16%), protein (13%), and fat (1.5%) in addition to riboflavin, thiamine, nicotinic acid, and minerals such as copper, manganese, magnesium, and selenium. These compounds exhibit significant biological activities, such as immune enhancement, anti‐aging, anti‐tumor effects, and antioxidant properties (Miguel 2022; Luo et al. 2014). Recent research indicates that berries exert beneficial effects on nutrition and human health, largely due to taste‐ and health‐related compounds such as organic acids, phenolic acids, and sugars (Ali, Ayub, et al. 2025; Wan et al. 2025; Stabnikova et al. 2024; Mikulic‐Petkovsek et al. 2012; Zheng et al. 2010).

Free sugars found in many fruits and vegetables are functional components that play a fundamental role in imparting flavor and aroma, maintaining fruit quality, and determining nutritional and nutraceutical value (Wu et al. 2012). In berries, free sugars are a predominant nutritional component closely linked to yield and quality, influencing growth, ripening, and composition (Zhao et al. 2015). The characteristics and concentrations of these components vary during ripening, determining changes in organoleptic properties and significantly affecting the quality of food products in the market (Muniyandi et al. 2025; Rodrigo et al. 2012).

Among the numerous bioactive compounds that contribute to the health‐promoting properties of *Lycium barbarum* (goji berries), polysaccharides (LBP) and total sugars play a particularly important role (Q. Lu et al. 2025; Tian et al. 2019; Zeng et al. 2023). Total sugars in goji berries consist of a mixture of mono‐, oligo‐, and polysaccharides, with fructose, glucose, and sucrose being the three main components in ripe fruit. These sugars not only contribute to the fruit's nutritional profile but are also closely linked to its functional, medicinal, and sensory properties. Sugars associated with sucrose metabolism, mainly glucose, fructose, and sucrose, are critical not only to the metabolic processes but also to fruit quality (Y.‐M. Lu and Zhang 2000). This prevalence of glucose and fructose is not unique to *L. barbarum* ; for example, in tomatoes, although sucrose is the main form of sugar transported, it represents only a small fraction of the total, while glucose and fructose together account for more than 50% of the water‐soluble sugars in ripe fruit (Montesano et al. 2016; Robinson et al. 1988).

Amino acids, which are also an essential part of a healthy diet, are the basic structural units of proteins and play a central role in metabolism, the nervous system, and the digestive system (Sarkadi 2019). In addition, some free amino acids are responsible for the flavor and taste of foods; for example, L‐glutamate elicits a unique taste called “umami” (Kondoh et al. 2009). Beyond their nutritional role, amino acids benefit physiological processes such as immunity modulation, anti‐arrhythmic, anti‐tumor, and anti‐viral effects (Guo et al. 2015; Liu et al. 2013). Goji berries contain 1.0%–2.7% free amino acids, the most abundant of which is proline. The presence of taurine and betaine has also been confirmed (Y. Lu et al. 2021; Potterat 2010). Together, sugars and amino acids not only define the nutritional and functional profile of goji berries but also shape their sensory attributes, ultimately influencing consumer preference. Understanding their dynamic changes during fruit development and under different environmental conditions is therefore vital for optimizing cultivation and harvest strategies.

Studies on goji berries ( *Lycium barbarum* L. and *L. chinense* Mill.) have also highlighted the significant influence of environmental conditions, climate, and harvest time on fruit quality and composition (Mi et al. 2020; Poggioni et al. 2022). Research conducted in Turkey, representing a transitional climate, and in central Italy, under a Mediterranean climate, has specifically investigated the effect of harvest time and growing season on goji berry characteristics (Poggioni et al. 2022; Polat et al. 2020). Pomological traits, such as fruit length, width, and weight, were generally found to be greater earlier in the production season and tended to decrease progressively toward the end of the season (Polat et al. 2020). Conversely, several key phytochemical characteristics tend to increase later in the harvest season (Poggioni et al. 2022; Polat et al. 2020).

Total phenolics, anthocyanins, soluble solids (SSC), and antioxidant activity generally increase as the season progresses. Polat et al. (2020) reported significant increases from the first to the last harvest in anthocyanins (264%), phenolics (48%), and antioxidant activity (105%), correlating SSC growth with environmental ripening conditions (Turkey). Conversely, Poggioni et al. (2022) found that while polyphenols and zeaxanthin peaked in September (Italy), flavonoid content and antioxidant power were highest at the beginning of the fruiting season. Beyond seasonal changes within a year, significant physical and chemical variations occur between growing seasons due to meteorological conditions. In China, goji berries show marked regional differences in morphology and composition (Yao et al. 2018), driven by a complex interplay of ecological factors such as altitude, humidity, temperature, soil potassium, and precipitation (Mi et al. 2020). These environment‐driven properties suggest that berries from different regions are optimally suited for specific purposes: large fruits for the fresh market, high‐sugar varieties for canning, and those with high antioxidant activity/bitterness for medicinal use (Yao et al. 2018).

Recent studies have further emphasized that climate‐driven stressors, particularly heat waves and drought, strongly modulate primary metabolites such as sugars and amino acids in many fruit species, including those cultivated in Mediterranean environments. However, integrated analyses of these metabolites in goji berries under Mediterranean conditions remain scarce (Medda et al. 2022).

This study aims to characterize the seasonal dynamics of free sugars and amino acids in organically grown *L. barbarum* berries in Southern Tuscany, evaluating how their concentrations vary throughout the fruiting season and how they relate to climatic conditions over three consecutive years. To capture these patterns, we analyzed ripe berries collected across the summer–autumn period and examined free sugar profiles at different ripening stages (S1–S4), allowing us to follow metabolic changes from fruit set to full maturity and to identify the most suitable harvest window. The novelty of this work lies in its integrated, multi‐year assessment of both sugars and amino acids in goji berries cultivated under Mediterranean conditions, combined with quantitative correlations to temperature and precipitation. By linking metabolite profiles with environmental variability and developmental stage, this study provides new insights into how climate influences goji berry composition and offers practical guidance for optimizing cultivation and harvest strategies.

## 2 Materials and Methods

### 2.1 Study Area

Samples were collected during three consecutive seasons (2018, 2019 and 2020) at the Bragaglia Organic Farm, one of the first to grow goji in Tuscany. The field is located in the Maremma hinterland at an altitude of approximately 250 m above sea level, near Magliano in Toscana (42°38′29.75”N, 11°16′17.45″ E). The plantation currently covers an area of 1 ha and contains approximately 1800 plants arranged in rows of approximately 130 m with a planting density of 1.6 m × 2.8 m.

### 2.2 Climatic Conditions

Climatic data during the fruiting seasons of the 3 harvest years were recorded by a public thermo‐pluviometric station of the Tuscan Region (SIR network, https://www.sir.toscana.it) located 5 km from the farm (Table 1). Table 1 reports climatic data for all fruiting months (July–November) to provide environmental context; however, no fruit samples were collected in August because plants did not produce sufficient material for representative sampling.

**TABLE 1:** Climatic data recorded over three consecutive years (2018–2020) in the goji cultivation area in southern Tuscany. The dataset includes monthly mean temperature (T) and total precipitation (P) for the fruiting months (July–November). August data are reported exclusively to describe the climatic context of the growing season; no fruit sampling was performed in August due to insufficient fruit production under high temperature and drought conditions.

			Jul	Aug	Sep	Oct	Nov	Fruiting season	Year
2018	P (mm)		4	77.4	47	87.6	136.6	352.6	966.8
	T (°C)	mean	25.5	25.9	22.5	18.4	13	21.1	16.3
		SD	±7.7	±7.8	±7.4	±6.0	±5.4	±6.9	±6.8
2019	P (mm)		30.6	1	82.4	114.8	291.6	520.4	916.8
	T (°C)	mean	26.1	26.4	22	18.4	13	21.2	16.2
		SD	±8.5	±8.4	±7.3	±6.2	±4.5	±7.0	±6.8
2020	P (mm)		6.4	46.2	65.8	82.2	54	254.6	536.2
	T (°C)	mean	25.8	27.3	22.1	14.7	13	20.6	16.2
		SD	±8.6	±8.3	±8.0	±5.8	±5.8	±7.3	±6.5

### 2.3 Fruit Sampling

Approximately 50 g of fruit was collected from the entire canopy at commercial maturity, intact and without alteration. The different pools were collected during 3 fruiting seasons: 1 sampling during the 2018 season, 7 samplings in 2019, and 8 samplings in 2020. In the 2019 and 2020 seasons, sampling was carried out at different time points during the fruiting period (July, September, October, November). In the month of August, no fruits were ever collected because the plants never provided suitable material for sampling. For each sampling point, three independent biological replicates were collected, each consisting of ~50 g of fruit sampled from different plants within the orchard.

Fruits were also selected to determine the free sugar profile at different stages of ripening (data not published). Here is a summary: stage 1 (S1) begins with the fruit set and lasts 3–5 days; fruits are hard and entirely green; stage 2 (S2) follows the S1 phase and lasts 2–4 days; fruits remain mainly green, with less than 50% of the surface showing orange coloration; stage 3 (S3), onset of nearly complete version; fruits exhibit prominent orange coloration; stage 4 (S4) marks complete ripening; fruits are deep orange‐red, soft, and free from deformation; this occurs around the 10th–12th day of development; fruits can be easily detached from the stalk.

After sampling, the fruits were powdered in liquid nitrogen and stored at −20°C until use.

### 2.4 Sugar Analysis

Free sugar content analyses were conducted on samples collected over a period of three consecutive years. For free sugar determination, 0.1 g of material was extracted with 1 mL of deionized Milli‐Q water. Samples were lysed with Ultra Turrax and the resulting solution was filtered through 0.45 μm filters, following a procedure adapted from Mikulic‐Petkovsek et al. (2012). Analysis was performed on a Waters 2487 IR HPLC with a C18 SUGAR‐PARK I ion exchange column (300 mm × 6.5 mm, Waters) packed with calcium cation exchange gel microparticles and thermostated at 90°C (Waters Corporation, n.d.‐a) The mobile phase consisted of 100% distilled water, and the flow rate was set at 0.5 mL/min. The injection volume was 20 μL with a run time of 20 min. The signal from the detector was converted to digital format using an A/D converter and managed by Clarity CSW‐32 software. Carbohydrate concentrations were calculated using reference curves constructed from certified standards with concentrations ranging from 0.1 to 10 mg/mL. Results were expressed as mg/g fresh weight.

### 2.5 Amino Acid Analysis

Amino acids content analyses were conducted on samples collected over a period of two consecutive years. Amino acids were determined fluorimetrically after prederivatization using the WATERS AccQ.Tag method for HPLC. The HPLC system consisted of a WATERS LC‐MODULE 1 and a 2475 fluorimetric detector, together with a 250 mm × 4.6 mm, 5 um C18 column, thermostated at 40°C (Waters Corporation, n.d.‐b). Samples and reference standards were treated according to the derivatization protocol described in the respective manual. A quantity of 0.1 g of material was extracted with 1 mL of Milli‐Q deionized water. Samples were lysed with Ultra Turrax and the resulting solution was filtered through 0.45 μm filters (adapted from Mikulic‐Petkovsek et al. 2012). The procedure involved adding 70 μL of borate buffer and 20 μL of fluorescent reagent to 10 μL of sample, resulting in a final volume of 100 μL. Standards were run in duplicate prior to each reading and treated as follows: 60 μL borate buffer and 20 μL fluorescent reagent were added to 10 μL protein amino acid standards and 10 μL non‐protein amino acid standards. Samples and standards were then incubated at 55°C for 15 min. A gradient consisting of a phosphate, sodium acetate and triethylamine buffer, pH 5 (A) and a 60% acetonitrile solution in MilliQ water (B) was used as the mobile phase: The data show that the first period is 0–0.84 min, with a 100% A; the second period is 0.84–25 min, with a 98% A; the third period is 25–31.70 min, with a 93% A; the fourth period is 31.70–53.40 min, with a 90% A; the fifth period is 53.40–61.80 min, with 67% A; the sixth period is 61.80–63.50 min, with 75% A; and the seventh period is 63.50–70 min, with 100% A. The injection volume was 5 μL of sample or standard, and the flow rate was set at 1.5 mL/min. The excitation wavelength was 250 nm and the emission wavelength was 395 nm.

The signal from the detector was converted to digital format using an A/D converter and managed by Clarity CSW‐32 software. Amino acid concentrations were calculated using reference curves constructed from certified standards with variable concentrations starting at 50 nmol/mL. The results were finally expressed in μg/g fresh weight.

### 2.6 Statistical Analysis

The data were analyzed to obtain means and standard deviations. Two‐way ANOVA was then performed using StatPlus (AnalystSoft Inc. 2021) software to evaluate the effects of year and month on metabolite concentrations because both factors were categorical and independent, and the dataset met the assumptions of normality and homoscedasticity. When significant main or interaction effects were detected (*p* ≤ 0.05), post hoc comparisons were performed using Bonferroni's correction to control for type I error inflation due to multiple testing. Pearson's correlation analysis was used to assess linear relationships between metabolite levels and climatic variables, as both types of data were continuous and normally distributed.

## 3 Results and Discussion

### 3.1 Sugar Profile Analysis

The sugar profile of goji berries reflects both their metabolic status and environmental adaptation during fruit development and ripening. This section explores the composition, temporal variation, and eco‐physiological correlations of major sugars and polysaccharides detected in the fruit, with comparative references to literature.

#### 3.1.1 Sugar Composition in Fully Ripe Berries

As shown in Table 2, fully ripe goji berries (S4) were characterized by a dominance of monosaccharides, with glucose (80.15 ± 19.30 mg/g FW) and fructose (75.49 ± 18.09 mg/g FW) as the most abundant sugars, significantly exceeding sucrose content (2.47 ± 1.94 mg/g FW). Both monosaccharides exhibited high variability, with concentrations ranging from approximately 36 to 123 mg/g FW. Pectins represented the third most abundant component, with a mean content of 46.30 mg/g FW (range: 16.69–105.97 mg/g FW). In contrast, minor sugars, including melezitose and maltohexaose, and sugar alcohols such as mannitol, xylitol, and sorbitol were detected only at low or trace concentrations (mean values ≤ 0.23 mg/g FW). Despite their low averages, some of these compounds, particularly mannitol, showed occasional high accumulations in specific samples. Finally, ethanol was detected with a mean concentration of 2.88 mg/g FW, showing peak values up to 15.93 mg/g FW. The data reported in Table 2 show a wide dispersion of values (expressed as standard deviation), reflecting the high metabolic heterogeneity of the berries sampled over the three‐year period. Such variability is indicative of the plasticity of *L. barbarum* in response to environmental fluctuations at the cultivation site and the intrinsic variability among different genotypes.

**TABLE 2:** Descriptive statistics of sugar, pectin, and ethanol concentrations (mg/g FW) in ripe goji berries ( *Lycium barbarum* L.). The total mean concentrations (±SD), minimum, maximum, and range are expressed relative to the sugars detected. The values are given in milligrams per gram of fresh weight (mg/g FW).

	Mean	SD	Min	Max	Range
Pectins	46.30	16.09	16.69	105.97	89.28
Sucrose	2.47	1.94	0.00	10.93	10.93
Glucose	80.15	19.30	35.69	122.85	87.16
Fructose	75.49	18.09	36.38	120.95	84.57
Melezitose	0.10	0.25	0.00	1.42	1.42
Maltohexaose	0.05	0.36	0.00	3.04	3.04
Mannitol	0.23	0.93	0.00	7.95	7.95
Sorbitol	0.11	0.72	0.00	4.90	4.90
Xilitol	0.21	0.83	0.00	4.67	4.67
Ethanol	2.88	2.98	0.00	15.93	15.93

Sugars are key quality determinants influencing fruit flavor and consumer acceptance, yet relatively few studies have examined the detailed sugar profile of *L. barbarum* berries (Kader 2008). In fully ripe fruits, glucose and fructose typically occur at comparable concentrations, a pattern also observed in the present study. However, published data show considerable variability. Zhao et al. (2015), for example, reported markedly higher fructose (40.70 ± 3.76 mg/g) than glucose (2.34 ± 0.06 mg/g), with sucrose remaining low (0.68 ± 0.03 mg/g) and minor sugars such as erythrose, galactose, and arabinose detected only in trace amounts (< 0.1 mg/g).

Studies on dried berries reveal even broader ranges. Montesano et al. (2016) found high fructose (154.20–259.13 mg/g) and glucose (152.92–284.60 mg/g) levels in commercial samples, while sucrose was approximately tenfold lower (13.75–36.43 mg/g). Similarly, Mikulic‐Petkovsek et al. (2012) reported 23.1 g/kg of both glucose and fructose and only 0.38 g/kg of sucrose in *L. barbarum* , values comparable to those obtained in this study. These differences likely reflect environmental influences, such as the cooler continental climate of Slovenia versus the Mediterranean conditions of the Tuscan coast. The same study also provided comparative data for other berry species. In cranberry, blackberry, strawberry, and red currant, glucose and fructose concentrations were nearly identical, whereas sucrose was consistently an order of magnitude lower. This pattern mirrors that of goji berries and supports the notion that hexoses dominate the sugar profile in most soft fruits. Ethanol, detected in small amounts, may arise from alcohol dehydrogenase activity during ripening under transient hypoxic conditions (Pesis 2005).

Climatic variables significantly affected sugar composition in ripe goji berries (Table S1). Precipitation showed positive correlations with pectin (*r* = 0.202) and sucrose (*r* = 0.551), while mean temperature was negatively associated with fructose and sucrose (*r* = −0.320* and −0.309**, respectively). These patterns suggest that cooler, wetter conditions promote sugar accumulation, possibly by reducing respiratory losses and modulating key enzymes of carbohydrate metabolism. Conversely, higher temperatures may limit sugar biosynthesis and favor carbon allocation toward alternative metabolic pathways. Such sensitivity to environmental variability aligns with previous findings in goji berries (Montesano et al. 2016).

To further explore how environmental factors shape sugar accumulation, we examined interannual trends across the three growing seasons.

#### 3.1.2 Inter‐Annual Variation (2018–2020)

**FIGURE 1:**
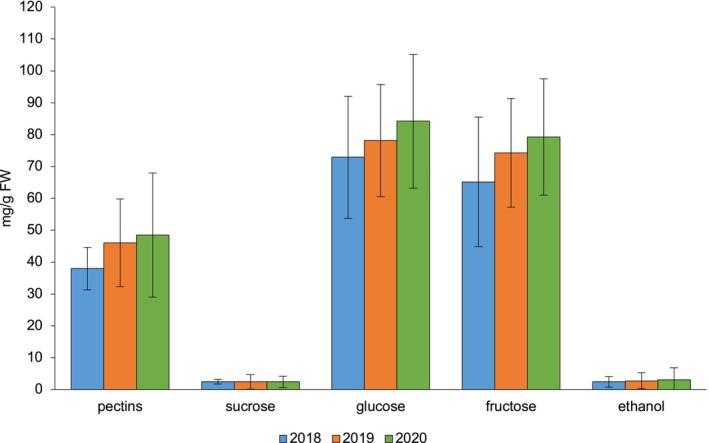
Annual trends in mean concentrations (±SD) of pectins, sucrose, glucose, fructose and ethanol in *L. barbarum* berries over three consecutive seasons (2018–2020). All values are reported in milligrams per gram of fresh weight (mg/g FW). Error bars represent the standard deviation (SD).

This section examines inter‐annual trends in sugar and pectin accumulation in goji berries over three consecutive growing seasons (2018–2020), with particular attention to potential correlations with temperature and rainfall patterns. Understanding these year‐to‐year fluctuations is essential for evaluating how climatic variability shapes fruit biochemical composition and overall crop quality. The temporal dynamics of sugar content during this period are illustrated in Figure 1.

Although no statistically significant differences emerged among years, glucose, fructose, and pectins displayed a consistent upward trend across the study period, suggesting a possible influence of long‐term climatic variation. Glucose increased from 72.91 ± 19.17 in 2018 to 84.22 ± 20.96 mg/g in 2020, and fructose from 65.12 ± 20.33 to 79.28 ± 18.28 mg/g. Pectin content rose from 37.97 ± 6.66 to 48.48 ± 19.51 mg/g. In contrast, sucrose remained stable across years (2.46–2.49 mg/g), while ethanol showed a modest increase (2.47 ± 1.65 in 2018 to 3.10 ± 3.71 mg/g in 2020).

The standard deviations observed, particularly during the late ripening stages (S3 and S4), are not a result of sampling imprecision but reflect the inherent biological variance of the species. The attribution of berries to the four ripening stages was based on strict, objective morphological and chromatic criteria (e.g., epicarp color and texture), ensuring high intra‐stage consistency. However, the scalar ripening behavior of *L. barbarum* , where berries on the same branch reach the same phenological stage at different metabolic rates, results in a natural physiological spectrum. Consequently, the observed variability represents an accurate and transparent snapshot of the real‐world metabolic diversity of the crop, rather than an experimental artifact.

These compositional shifts align with the climatic context of the study area: 2018 was the wettest year, whereas 2020 experienced the lowest precipitation, approximately half that of 2018 and the driest fruiting season. Reduced rainfall and higher evaporative demand may have favored hexose accumulation and pectin concentration through osmotic adjustment and altered carbon allocation.

Similar inter‐annual variability has been documented in other fruit crops. In blueberries, temperature, precipitation, and altitude exerted stronger effects on sugar accumulation than cultivar identity, with marked year‐to‐year fluctuations reported for all major sugars (Correia et al. 2016). In plums, harvest year significantly influenced sucrose, sorbitol, total sugars, and dry matter, while glucose and fructose were more strongly determined by genotype (Cupic et al. 2018).

The consistently low sucrose levels observed in goji berries, as also reported for blueberries, may reflect rapid hydrolysis or conversion into hexoses during ripening. Fructose, over 1.5 times sweeter than sucrose and the most water‐soluble natural sugar, plays a central role in determining fruit sweetness, whereas glucose, although less sweet, is a key metabolic substrate and precursor for essential biomolecules (Hanover and White 1993).

#### 3.1.3 Monthly Trends During Fruiting Season

Seasonal variation during the harvest period can significantly affect the biochemical composition of fruits. To explore these dynamics in *L. barbarum* , we examined monthly changes in sugar and pectin content across four representative harvest months (July, September, October, and November; Table 3), with the aim of identifying patterns linked to ripening progression and environmental conditions.

**TABLE 3:** Monthly variations in mean concentrations (±SD) of main sugars and pectins (mg/g FW) in goji berries. Mean concentrations (±SD) of the main sugars detected in the months of fruiting (July, September, October and November). The values are expressed in milligrams per gram of fresh weight (mg/g FW). Different lowercase letters within a row indicate significant statistical differences between months (July–November) according to the Bonferroni post hoc test (*p* < 0.01). The high standard deviations observed reflect the intrinsic biological variability and the dynamic response of fruit carbohydrates to fluctuating field microclimates.

	July	September	October	November
Pectins	44.97 ± 10.30^a^	39.61 ± 12.79^a^	48.13 ± 18.22^a^	64.27 ± 21.93^b^
Sucrose	1.99 ± 1.33	2.01 ± 1.55	3.20 ± 2.40	3.74 ± 2.68
Glucose	73.06 ± 15.03	84.33 ± 21.27	86.91 ± 12.08	78.42 ± 22.37
Fructose	69.03 ± 15.33	81.18 ± 21.03	80.21 ± 18.11	71.94 ± 11.35
TSC	189.05 ± 41.99	204.13 ± 56.64	218.45 ± 50.81	218.37 ± 58.33

Among the analyzed compounds, only pectins showed statistically significant differences across months, with a pronounced increase in November (64.27 ± 21.93 mg/g) compared with July (44.97 ± 10.30 mg/g), September (39.61 ± 12.79 mg/g), and October (48.13 ± 18.22 mg/g). This rise suggests intensified cell wall remodeling during late‐season development. Although sucrose, glucose, and fructose did not vary significantly, their temporal profiles revealed consistent trends. Sucrose gradually increased toward the end of the season, from 1.99 ± 1.33 mg/g in July to 3.20 ± 2.40 mg/g in October and 3.74 ± 2.68 mg/g in November. Glucose and fructose peaked in September and October (84.33 ± 21.27 and 86.91 ± 12.08 mg/g for glucose; 81.18 ± 21.03 and 80.21 ± 18.11 mg/g for fructose), followed by a slight decline in November. These patterns indicate that sugar accumulation remains relatively stable throughout the fruiting season, whereas pectin content is more responsive to late‐ripening processes, potentially linked to tissue softening and over‐ripening.

Total sugar content (TSC), including pectins, glucose, fructose, and sucrose, increased from July (189.05 ± 41.99 mg/g) to October (218.45 ± 50.81 mg/g) and remained comparable in November (218.37 ± 58.33 mg/g), although without statistical significance. Overall, these results suggest that within a single season, hexose accumulation follows a relatively uniform trajectory, while pectin dynamics provide a more sensitive indicator of advanced ripening stages. Changes occurring during late ripening often involve cell wall remodeling processes that affect the physical behavior of fruit tissues, largely driven by modifications in pectic components (Ali, Nizigiyimana, et al. 2025), although no direct rheological measurements were performed in the present study.

As shown in Table 3, the standard deviations associated with the monthly means are marked. This variability reflects the dynamic nature of the fruits’ physiological responses to the shifting microclimatic conditions (temperature and radiation) that characterize the long fruiting period, from July to November, within the Mediterranean context.

Similar patterns have been observed in other species. For example, Zorenc et al. (2016), in a study on blueberries, reported a general increase in total sugar content over the harvest period. Interestingly, berries harvested later in the season, despite being smaller in size, contained comparable or even higher concentrations of key metabolites than earlier‐harvested, larger fruits. These findings support the notion that metabolic maturity can proceed independently of fruit size and that sugar accumulation may persist into the late stages of the fruiting season.

#### 3.1.4 Changes During Ripening Stages (S1–S4)

The ripening process in fleshy fruits is typically associated with dynamic modifications in sugar metabolism and cell wall architecture. This section explores the progression of key sugars and pectins across four distinct developmental stages (S1–S4) in *L. barbarum* , highlighting the main biochemical transformations that characterize fruit maturation.

**FIGURE 2:**
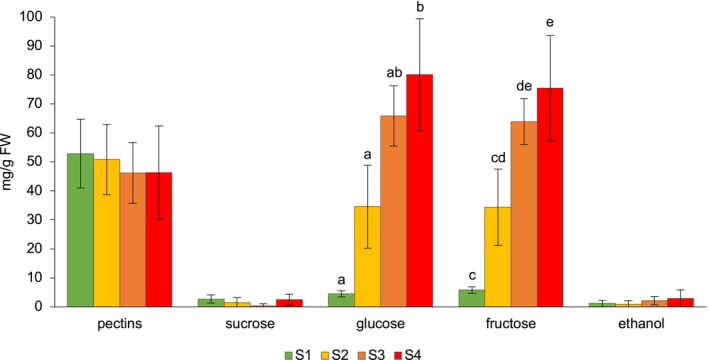
Dynamic evolution of pectins, sugars, and ethanol concentrations across four progressive ripening stages (S1–S4) in *L. barbarum* berries. Concentrations are expressed in milligrams per gram of fresh weight (mg/g FW). Different letters above the bars indicate significant differences between stages (*p* < 0.01) based on the Bonferroni post hoc test. Error bars represent the standard deviation (SD).

Marked changes in sugar composition were observed during ripening, particularly for glucose and fructose (Figure 2). At the initial stage (S1), the concentrations of sucrose, glucose, and fructose were relatively low and comparable (2.68 ± 1.44, 4.51 ± 1.09, and 5.79 ± 1.14 mg/g, respectively). A sharp increase occurred between S1 and S2, with glucose and fructose rising to 34.54 ± 14.36 and 34.32 ± 13.23 mg/g. Sucrose decreased slightly but without statistical significance.

From S2 onward, glucose and fructose continued to accumulate, reaching maximum values in S4 (80.15 ± 19.30 and 75.50 ± 18.09 mg/g, respectively). Statistically significant differences were observed between the early (S1–S2) and late stages (S3–S4), while the absence of differences between S3 and S4 suggests a stabilization phase in hexose accumulation. Sucrose remained consistently low throughout ripening (range = 4.16 mg/g), with significant differences detected only between S3 and S4, confirming its minor role in the overall sugar profile.

Pectin concentrations showed limited variation across stages (52.82 ± 11.86 mg/g in S1 to 46.30 ± 16.09 mg/g in S4), indicating that major cell wall modifications may occur earlier or in a more localized manner. Ethanol displayed an increasing trend (1.11 ± 1.12 mg/g in S1 to 2.88 ± 2.98 mg/g in S4), although differences were not statistically significant, suggesting low fermentative activity under the conditions tested. Among minor sugars, melezitose was the only compound consistently detected across ripening stages (0.46–1.45 mg/g). Its presence may relate to osmotic regulation or ecological interactions, such as insect attraction, as reported in other species like pineapple, where melezitose increases during ripening to support insect‐mediated seed dispersal (Ikram et al. 2020). The large standard deviations visible in Figure 2, especially for glucose and fructose during the 2020 season, appear to be correlated with the physiological stress potentially induced by the extreme drought recorded that year. Such conditions may have intensified intra‐sample variability in carbon allocation responses and fruit osmoregulation processes.

The progressive accumulation of glucose and fructose observed here aligns with previous findings in goji berries (Zheng et al. 2010) and in other fruit species, including apples, strawberries, apricots, plums, and peaches (Zhang et al. 2010; Basson et al. 2010; Bae et al. 2014). However, contrasting trends have been reported in jujube (Song et al. 2019) and in the strawberry cultivar “Camarosa” (Kafkas et al. 2007), highlighting species‐specific metabolic strategies. Ethanol accumulation during ripening has also been documented in peach and nectarine cultivars (Zerbini et al. 2001), supporting its potential role as an indicator of advanced maturity.

### 3.2 Free Amino Acid Profile Analysis

Amino acids in goji berries serve both nutritional and functional roles, contributing to protein synthesis, stress adaptation, and fruit quality. This section presents the general amino acid composition, temporal variability, and their relationship with environmental stress, with emphasis on key regulatory compounds such as proline and BABA.

#### 3.2.1 Free Amino Acids Composition in Fully Ripe Berries

In this study, 23 free amino acids (AAs) were identified, totaling a mean concentration of 3208.12 ± 1239.77 μg/g FW (Table 4). Proteinogenic AAs were predominant (2872.21 ± 1105.86 μg/g), primarily composed of non‐essential amino acids (NEAAs; 55.4%) followed by essential amino acids (EAAs; 24.8%). Within the NEAAs, proline (PRO) was the most abundant (1045.91 ± 657.30 μg/g), followed by serine (SER; 398.20 ± 373.89 μg/g) and glutamic acid (GLU; 144.17 ± 69.28 μg/g). Other proteinogenic NEAAs, such as aspartic acid, glycine, and tyrosine, were present at moderate levels, while cysteine remained the least represented. Among EAAs, arginine (ARG) was the most represented (381.07 ± 267.95 μg/g), followed by histidine and leucine. Other EAAs, including threonine and isoleucine, showed intermediate levels, whereas methionine, lysine, and phenylalanine were detected only in trace amounts (< 25 μg/g). Non‐proteinogenic amino acids (NPAAs), though less abundant (335.92 ± 394.45 μg/g), included physiologically significant compounds such as alanine (297.18 ± 175.16 μg/g) and γ‐aminobutyric acid (GABA; 161.53 ± 351.31 μg/g). GABA, in particular, exhibited high variability (up to 1458.79 μg/g), reflecting its role in stress response. Other NPAAs, such as taurine, ornithine, and β‐aminobutyric acid, were detected at lower or baseline concentrations, yet remain relevant for their roles in nitrogen metabolism and plant defense.

**TABLE 4:** Total mean values (±SD), minimum, maximum and range related to the 23 amino acids detected, expressed in micrograms per gram of fresh weight (μg/g FW).

	Mean	SD	Min	Max	Range
Non‐essential AA					
ASP	132.85	90.67	40.05	396.55	356.50
SER	398.20	373.89	72.62	1343.44	1270.82
GLU	144.17	69.28	70.39	288.65	218.27
GLY	33.91	25.86	0.00	100.70	100.70
PRO	1045.91	657.30	0.00	2330.05	2330.05
CYS	2.42	9.07	0.00	36.35	36.35
TYR	20.47	8.51	4.69	34.05	29.35
Essential AA					
HYS	110.80	258.36	0.00	901.91	901.91
ARG	381.07	267.95	0.00	988.83	988.83
THR	69.35	124.63	0.00	433.92	433.92
VAL	79.32	28.26	34.29	138.91	104.62
MET	1.37	1.12	0.00	4.30	4.30
ILE	22.15	17.75	0.00	61.43	61.43
LEU	109.51	54.07	0.00	205.58	205.58
LYS	14.92	10.72	0.00	31.97	31.97
PHE	8.62	6.97	0.00	20.49	20.49
Non‐proteogenic AA					
TAU	78.20	64.47	0.00	268.06	268.06
BALA	59.49	118.96	0.00	351.70	351.70
ALA	297.18	175.16	0.00	615.73	615.73
GABA	161.53	351.31	0.00	1458.79	1458.79
BABA	0.12	0.34	0.00	1.03	1.03
AABA	16.65	28.33	0.00	95.37	95.37
ORN	19.92	12.47	1.19	45.87	44.68
TOT	3208.12	1239.77	—	—	—
Proteogenic AA	2872.21	1105.86	—	—	—
Non‐proteogenic AA	335.92	394.45	—	—	—
Essential AA	797.10	404.42	—	—	—
Non‐essential AA	1777.93	650.93	—	—	—

Amino acids play multiple essential roles in plant metabolism. Beyond serving as protein building blocks, they act as precursors for secondary metabolites, intermediates in biosynthetic pathways, and regulators of physiological processes such as ion transport, stomatal function, gene expression, and enzyme activity (Kumar et al. 2017; Yang et al. 2020). Their involvement in redox homeostasis and osmotic regulation is crucial under abiotic stress conditions, where compounds such as proline (PRO) accumulate to stabilize cellular structures and mitigate oxidative damage (Hayat et al. 2012). Some AAs, like glutamic acid (GLU), also contribute to flavor development (umami taste), and many are nutritionally important in human diets (Galili et al. 2016).

The dominance of NEAAs, particularly PRO and SER, in *L. barbarum* fruit confirms their key role in stress‐related metabolic pathways. The substantial variability observed in PRO and GABA concentrations suggests that their accumulation is highly responsive to environmental and developmental factors. The consistent presence of ORN among NPAAs further supports the relevance of nitrogen‐associated metabolism in these fruits.

When compared with literature data, the absolute concentrations measured in this study are considerably lower. Y. Lu et al. (2021) reported total AA contents ranging from 1020 to 2584 mg/100 g FW (approximately tenfold higher), while Zhou et al. (2020) found 24.57 ± 1.08 mg/g FW across 29 AAs. Similar higher ranges have been documented in tomato (16.82–32.79 mg/g FW; S. H. Choi et al. 2014), jujube (1.29–40.68 mg/g FW; S.‐H. Choi et al. 2012), Hami melon juice (3.40–4.70 mg/g FW; Pei et al. 2020), and apple (0.25–3.20 mg/g FW; Di Maro et al. 2011). Despite the lower total AA concentrations, *L. barbarum* berries exhibit a unique profile rich in stress‐related and nutritionally valuable amino acids physiological mechanisms.

Having established the baseline amino acid composition, we next explored how these profiles varied across years as underscoring their potential as a functional food ingredient and as a model for studying best harvest months.

#### 3.2.2 Inter‐Annual Variation (2019–2020)

To evaluate the impact of inter‐annual environmental variability on free amino acid content in *L. barbarum* berries, a two‐way ANOVA was performed to compare the 2019 and 2020 seasons (Table 5). Although total amino acid (AA) content did not differ significantly between years (*p* = 0.196), berries harvested in 2019 consistently showed higher average concentrations across all amino acid classes. Total AA content reached 3525.27 ± 1331.17 μg/g FW in 2019 compared with 2679.55 ± 940.77 μg/g FW in 2020. A similar pattern was observed for proteinogenic amino acids (PAA: 916.72 vs. 597.75 μg/g) and non‐proteinogenic amino acids (NPAA: 3098.04 vs. 2495.82 μg/g). The ANOVA analysis shows significant fluctuations in standard deviations between the 2 years (2019 and 2020). The high variance observed in 2019 suggests a more heterogeneous metabolic response of the nitrogenous fraction in the presence of higher rainfall compared to the following season.

**TABLE 5:** Two‐way ANOVA results and mean concentrations expressed in micrograms/g per fresh weight (μg/g FW ± SD) of amino acid fractions in *L. barbarum* berries.

	AA tot		PAA		EPAA		Non‐EPAA		Non‐PAA	
	Mean	SD	Mean	SD	Mean	SD	Mean	SD	Mean	SD
**2019**	3525.27	1331.17	916.717	452.191	1893.02	688.191	427.231	479.415	3098.04	1189.65
**2020**	2679.55	940.771	597.752	215.208	1586.10	589.896	183.732	92.703	2495.82	921.557
F_Y_	1.843		2.578		0.824		1.474		1.121	2.578
*p‐value*	0.196		0.131		0.379		0.245		0.308	0.131
jul	2681.32	1369.58	714.2	428.255	1336.63	495.494	435.16	544.113	2246.16	1023.66
sep	3810.54	1014.76	930.177	428.509	2246.96	545.934	248.605	136.5	3561.94	933.569
oct	3508.08	446.213	729.51	284.766	2136.01	111.419	200.895	85.397	3307.19	531.61
F_M_	1.61		0.485		6.325		0.482		3.457	
*p‐value*	0.237		0.626		0.012*		0.628		0.063	
F_YxM_	1.063		0.347		2.699		3.83		1.565	
*p‐value*	0.381		0.715		0.116		0.06		0.256	

*Note:* Data refer to total (AA tot), protein (PAA), essential (EPAA), non‐essential (non‐EPAA), and non‐protein (Non‐PAA) amino acids in fruits cultivated in southern Tuscany (2019–2020) across three harvest months (July, September, October). Asterisks (*) denote significant effects of harvest year (FY), month (FM), or their interaction (FY×M) at *p* < 0.05.

Among individual compounds, valine (VAL) and ornithine (ORN) exhibited significant inter‐annual differences (*p* < 0.01), with higher concentrations in 2019. This suggests that these compounds may be particularly sensitive to climatic or agronomic variability. The VAL values observed here are consistent with those reported by Y. Lu et al. (2021), who documented ranges of 6.06–19.67 mg/100 g.

ORN also showed a significant positive correlation with rainfall (*r* = 0.426*, see Table S1) and lower concentrations in 2020, a year marked by reduced precipitation, especially during the fruiting months. These findings align with the known involvement of ORN in the biosynthesis of polyamines and proline via the ornithine decarboxylase (ODC) and ornithine δ‐aminotransferase (δ‐OAT) pathways, both of which are activated under salinity and water‐stress conditions (Kalamaki et al. 2009).

#### 3.2.3 Monthly Trends During Fruiting Season

Seasonal trends in amino acid accumulation were evaluated over the fruiting months (July, September, and October; Table 5). Total AA content increased markedly from 2681.32 μg/g FW in July to a peak of 3810.54 μg/g FW in September, followed by a slight decline in October (3508.08 μg/g FW). Although these differences were not statistically significant at the total level (*p* = 0.237), more pronounced seasonal patterns emerged for specific amino acids.

**FIGURE 3:**
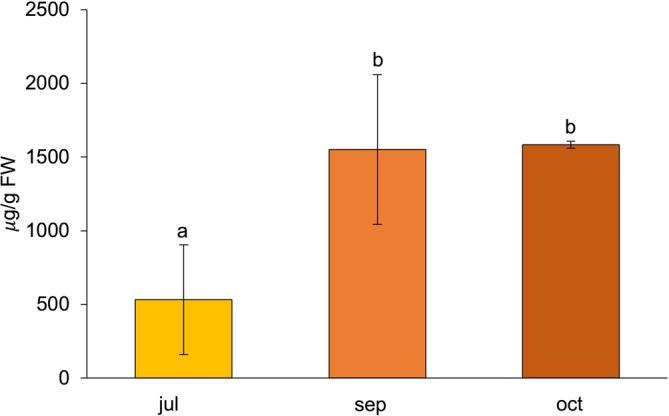
Monthly mean Proline (PRO) concentrations in *L. barbarum* berries during the fruiting season (July, September, and October). Values are expressed in micrograms per gram of fresh weight (μg/g FW). Different letters indicate significant monthly differences according to the Bonferroni post hoc test (*p* < 0.01). Error bars represent the standard deviation (SD).

Proline (PRO) exhibited the most marked variation (Figure 3), rising from 532.49 ± 372.85 to 1551.40 ± 507.52 μg/g in September, a statistically significant increase (*p* < 0.01). This peak coincides with the warmest and driest period of the season, conditions typically associated with osmoprotective responses in plants. The relatively large standard deviations observed across months likely reflect the strong heterogeneity in plant‐level stress exposure within the orchard, where differences in microclimatic conditions, fruit load, and canopy architecture can lead to highly variable PRO accumulation among individual berries. Such variability is common for stress‐responsive metabolites, whose synthesis is rapidly and non‐uniformly activated in response to fluctuating environmental cues.

PRO also showed a strong negative correlation with mean temperature (*r* = −0.652**) and a positive correlation with precipitation (Table S1), indicating that its synthesis is modulated by environmental stress and favored under cooler, wetter conditions. These trends are consistent with its multifunctional role in osmotic adjustment, redox regulation, and stress‐responsive gene expression (Trovato et al. 2019; Yang et al. 2020).

Conversely, β‐aminobutyric acid (BABA) levels remained low in July and September but increased significantly in October (0.99 ± 0.06 μg/g FW). This late‐season accumulation, coupled with a strong negative correlation with temperature (*r* = −0.862**, Table S1), suggests a role in defense activation and stress adaptation during the final stages of fruit development. BABA is known to induce systemic tolerance to multiple abiotic stresses, including drought, heat, salinity, and osmotic stress, through the activation of defense responses (Choudhary et al. 2021; Jakab et al. 2005; Jisha and Puthur 2016; Zimmerli et al. 2008). Its accumulation during the cooler end‐of‐season period suggests its role in the late‐phase stress adaptation of goji berries.

To further explore the combined effects of year and month, a two‐way ANOVA was performed (Table 5). While no significant interaction was detected for total AAs (*p* = 0.381), interaction trends emerged for specific categories: essential AAs (EPAA) approached significance (*p* = 0.116), and non‐proteinogenic AAs showed a marginally significant interaction (*p* = 0.060). These patterns suggest that amino acid accumulation may be shaped by the interplay between environmental conditions and phenological stage.

## 4 Conclusions

This study provides the first comprehensive characterization of free sugars and amino acids in *L. barbarum* berries cultivated in Italy, detailing their dynamics across ripening stages, seasons, and environmental conditions. The predominance of glucose and fructose at full ripeness, alongside the identification of minor sugars and polyols such as melezitose, mannitol, and sorbitol, highlights their dual role in enhancing both the berries’ nutritional value and stress tolerance.

Among amino acids, proline, BABA, and ornithine showed marked sensitivity to seasonal and climatic fluctuations, emerging as potential biomarkers of environmental stress and metabolic plasticity. Their behavior highlights the relevance of these compounds for eco‐physiological studies and for developing adaptive agronomic strategies.

Overall, the results emphasize the importance of tailored cultivation practices, including optimal harvest timing after S2 and the selection of genotypes capable of maintaining high hexose‐to‐sucrose ratios under variable climatic conditions. The metabolic fingerprints generated here support product authentication and nutraceutical standardization, reinforcing the need for quality traceability. The validated analytical framework also provides a basis for future research on genetic determinants of key metabolic traits and on compositional variability across different terroirs, ultimately contributing to improved fruit quality, market value, and cultivar identity in the nutraceutical sector.

## Author Contributions

**Giampiero Cai:** writing – review and editing, project administration.

## Funding

The authors have nothing to report.

## Ethics Statement

This study did not involve any human or animal subjects, and therefore no ethical approval was required.

## Consent

The authors have nothing to report.

## Conflicts of Interest

The authors declare no conflicts of interest.

## Supporting information


**Table S1:** Pearson correlation coefficients between sugars, AA and climate parameters (max rainfall in mm—Max R—and mean temperature—Mean T). Only significant correlations are shown (*p < 0,05; **p < 0,01).

## Data Availability

The data that support the findings of this study are available from the corresponding author upon reasonable request.
