# Supplementary material for: Seasonal and Environmental Influences on Free Sugar and Amino Acid Profiles of Lycium barbarum Berries Cultivated in Southern Tuscany
Source: Food Sci Nutr. 2026 Feb 19;14(2):e71568. doi: 10.1002/fsn3.71568 (PMC12920261; doi:10.1002/fsn3.71568)
Supplement: Supplementary file 1 — Table S1: Pearson correlation coefficients between sugars, AA and climate parameters (max rainfall in mm—Max R—and mean temperature—Mean T). Only significant correlations are shown (*p < 0,05; **p < 0,01). [file FSN3-14-e71568-s001.docx]

**Table S1.** Pearson correlation coefficients between sugars, AA and climate parameters (max rainfall in mm - Max R - and mean temperature - Mean T). Only significant correlations are shown (**p <0,05; **p<0,01*).

|  | **Pectins** | **Sucrose** | **GLY** | **ALA** | **PRO** | **BABA** | **ORN** | **LEU** | **nEAA** | **pAA** | **AAtot** |
| --- | --- | --- | --- | --- | --- | --- | --- | --- | --- | --- | --- |
| **Max R** |  | 0,202* |  | 0,551** | 0,776** |  | 0,426* |  | 0,806** | 0,744** | 0,635** |
| **Mean T** | -0,309** | -0,320** | -0,545** | -0,536* | -0,652** | -0,862** |  | 0,502* | -0,543* |  |  |
